# The MS-lincRNA landscape reveals a novel lincRNA BCLIN25 that contributes to tumorigenesis by upregulating ERBB2 expression via epigenetic modification and RNA–RNA interactions in breast cancer

**DOI:** 10.1038/s41419-019-2137-5

**Published:** 2019-12-04

**Authors:** Shouping Xu, Hongbo Liu, Lin Wan, Weijia Zhang, Qin Wang, Shumei Zhang, Shipeng Shang, Yan Zhang, Da Pang

**Affiliations:** 1 Department of Breast Surgery, Harbin Medical University Cancer Hospital, Harbin, HRB, 150040 China; 2 College of Bioinformatics Science and Technology, Harbin Medical University, Harbin, HRB 150081 China; 3 Department of Medicine, Icahn School of Medicine at Mount Sinai, New York, NY 10029 USA; 4 School of Life Science and Technology, Harbin Institute of Technology, Harbin, HRB 150001 China; 5 Heilongjiang Academy of Medical Sciences, Harbin, HRB, 150086 China

**Keywords:** RNA sequencing, Breast cancer

## Abstract

The landscape of molecular subtype-specific long intergenic noncoding RNAs (MS-lincRNAs) in breast cancer has not been elucidated. No study has investigated the biological function of BCLIN25, serving as a novel HER2 subtype-specific lincRNA, in human disease, especially in malignancy. Moreover, the mechanism of BCLIN25 in the regulation of ERBB2 expression remains unknown. Our present study aimed to investigate the role and underlying mechanism of BCLIN25 in the regulation of ERBB2 expression. The transcriptional landscape across five subtypes of breast cancer was investigated using RNA sequencing. Integrative transcriptomic analysis was performed to identify the landscape of novel lincRNAs. Next, WEKA was used to identify lincRNA-based subtype classification and MS-lincRNAs for breast cancer. The MS-lincRNAs were validated in 250 breast cancer samples in our cohort and datasets from The Cancer Genome Atlas and Gene Expression Omnibus. Furthermore, BCLIN25 was selected, and its role in tumorigenesis was examined in vitro and in vivo. Finally, the mechanism by which BCLIN25 regulates ERBB2 expression was investigated in detail. A total of 715 novel lincRNAs were differentially expressed across five breast cancer subtypes. Next, lincRNA-based subtype classifications and MS-lincRNAs were identified and validated using our breast cancer samples and public datasets. BCLIN25 was found to contribute to tumorigenesis in vitro and in vivo. Mechanistically, BCLIN25 was shown to increase the expression of ERBB2 by enhancing promoter CpG methylation of miR-125b, leading to miR-125b downregulation. In turn, ERBB2 mRNA degradation was found to be abolished due to decreased binding of miR-125b to the 3’-untranslated region (UTR) of ERBB2. These findings reveal the role of novel lincRNAs in breast cancer and provide a comprehensive landscape of breast cancer MS-lincRNAs, which may complement the current molecular classification system in breast cancer.

## Background

Breast cancer is the leading cause of death among women worldwide^1,2^. Previous findings have identified key protein-coding genes that are associated with breast cancer, such as BRCA1 and BRCA2, which are mutated in a subset of patients^3^. However, most breast cancer patients lack these genetic aberrations. Clinical studies have revealed that breast cancer is a heterogeneous disease at molecular, histopathological, and clinical levels^4–7^. At the clinical level, breast cancer is classified into five main subtypes [luminal A, luminal B (HER2+), luminal B (HER2−), HER2, and triple negative] based on immunohistochemical assays for estrogen receptor (ER), progesterone receptor (PR), human epidermal growth factor receptor 2 (HER2), and Ki-67^8^. Although classification based on breast cancer subtypes facilitates more precise tailoring of treatment approaches, the current subtyping system is still far from perfect. For example, patients with the same subtype according to the current subtyping system might react differently to the same drugs. Thus, the identification of novel biomarkers for multiple subtypes of breast cancer is required to complement the current subtyping system.

Recent studies have revealed that long intergenic noncoding RNAs (lincRNAs) are key regulators of diverse cellular processes, including development and tumorigenesis^9–11^. In addition, dynamic changes in lincRNA expression have been found in multiple cancers at various stages of disease^12,13^. For example, White et al. identified 111 differentially expressed lincRNAs in lung cancer using publicly available transcriptome sequencing data^14^. Accumulating evidence highlights the potential utility of lincRNAs as biomarkers and therapeutic targets in cancer^15,16^. For example, the use of the lincRNA biomarker PCA3 has been extensively investigated and successfully applied in clinical practice to predict biopsy outcomes in patients with elevated serum prostate-specific antigen expression. As important family members of long noncoding RNAs, lincRNAs can regulate the transcriptional levels of target genes and are strongly associated with cancer progression^17^. SChLAP1, a lincRNA corresponding to the most highly overexpressed gene in metastatic prostate cancer, is regarded as a potential biomarker for the prognosis of aggressive prostate cancer and as an indicator of the need for treatment intensification^18^. Furthermore, copy numbers of the lincRNA PVT1 are increased in more than 98% of cancers that have increased copy numbers of MYC, and high expression levels of PVT1 are associated with a poor prognosis in various cancer patients^19,20^. Thus, the identification of differential expression of lincRNAs has the potential to aid cancer diagnosis, treatment selection, and prognostic prediction^11^.

The relationship between lincRNAs and breast cancer has been reported in recent studies. Ding et al. identified 538 lincRNAs that were differentially expressed in breast cancer tissues but did not report their differential expression in different subtypes^21^. The expression of HOTAIR is dysregulated in many types of cancer, including breast cancer^22^. Merry et al. identified three lincRNAs that are dysregulated in response to ERBB2 amplification and result in the enhancement of breast cancer tumorigenesis^23^. LincRNAs have also been reported to function as important regulators of epithelial to mesenchymal transition, an event that promotes breast cancer progression and metastasis^24^. Despite the observation that breast cancer is a complex heterogeneous disease, only 18 breast cancer-related lincRNAs have been annotated in the LincRNADisease database for long noncoding RNA-associated diseases^25^. Together, these results highlight the significance of identifying and characterizing a comprehensive map of lincRNAs that are associated with breast cancer.

Whole transcriptome profiling using RNA sequencing (RNA-Seq) makes it possible to identify novel lincRNAs on a genome-wide scale. To identify novel lincRNAs in breast cancer, we carried out whole transcriptome sequencing of five molecular subtypes of breast tumors. By analyzing these transcriptomes, we identified novel lincRNAs, characterized their functional roles in the regulation of breast cancer and evaluated their association with distinct breast cancer subtypes. In particular, we established a novel breast cancer subtype classification system, which we termed Linctype, including molecular subtype-specific lincRNAs (MS-lincRNAs) and coding genes. These Linctype genes can not only distinguish breast cancer tissues from normal tissues but also classify breast cancer samples into five distinct molecular subtypes associated with significant prognostic differences. Independent qPCR validation in our cohort and analysis of data from the Cancer Genome Atlas (TCGA) and Gene Expression Omnibus (GEO) confirmed the differential expression patterns of these MS-lincRNAs in a large number of breast cancer tissues. Finally, as a HER2 subtype-specific lincRNA, BCLIN25 was selected, and its biological function and contribution to tumorigenesis were investigated in vitro and in vivo. Mechanistically, BCLIN25 was found to increase the expression of ERBB2 by enhancing the promoter CpG methylation of miR-125b, leading to the downregulation of miR-125b. In turn, ERBB2 mRNA degradation was abolished due to decreased binding of miR-125b to the 3’-untranslated region (UTR) of ERBB2. This study thus represents an integrated analysis of lincRNA and mRNA expression profiles in human breast cancer subtypes and provides a novel approach for the molecular stratification of breast cancer patients. Our results indicate that lincRNAs can be used for breast cancer molecular classification and may significantly complement the current subtyping system for breast cancer. Thus, the results presented in this study provide new insights into the molecular classification of breast cancer and open new avenues for the investigation of functional lincRNAs involved in breast cancer.

## Materials and methods

### Patient samples

Breast cancer and adjacent noncancerous tissues were obtained from patients at the Department of Breast Surgery, Harbin Medical University Cancer Hospital. Three normal breast tissues were obtained from patients undergoing breast reconstruction surgery. The tissues were divided into two groups following macroscopic review by two trained pathologists. One-half of each sample was immediately frozen in liquid nitrogen and stored at −80 °C until RNA extraction. The other half of each sample was fixed in buffered formalin and embedded in paraffin for hematoxylin-eosin staining. Only tumors consisting of >80% tumor cells were selected for RNA extraction. The molecular subtype of each cancer specimen was independently reviewed by two pathologists. The study protocol conformed to clinical research guidelines and was approved by the Research Ethics Committee of Harbin Medical University Cancer Hospital. Written informed consent was obtained from all patients who participated in this study.

### Library preparation for lincRNA sequencing

Total RNA was extracted using the AllPrep RNA Mini Kit (Qiagen, Hilden, Germany). Approximately 3 µg of RNA per sample was used for subsequent RNA sample preparations. Ribosomal RNA was removed using Human, Rat, and Mouse Epicentre Ribo-Zero™ Gold Kits (Epicentre, USA). Sequencing libraries were generated following the manufacturer’s recommendations, and varied index labeling was performed using the NEBNext® Ultra™ Directional RNA Library Prep Kit for Illumina (NEB, Ipswich, USA). Briefly, ribosomal RNA was extracted using the indicated kits, and RNA fragments were reverse transcribed using NEBNext First Strand Synthesis Reaction Buffer at an elevated temperature. The first cDNA strand was synthesized from RNA samples using random hexamers. Second cDNA strand synthesis was performed using buffer, dNTPs, DNA polymerase I and RNase H. The resulting DNA library was purified using the QIAquick PCR Kit and eluted with EB buffer, and terminal-repair poly (A) and adapter sequences were subsequently added. To preferentially select cDNA fragments that were 200 bp in length, we purified the library fragments by agarose gel electrophoresis. Additionally, the UNG enzyme was used to digest the second cDNA strand. The DNA fragments were amplified using PCR, and products of ~200 bp in length were recovered from agarose gels to complete library preparation. The libraries were then sequenced on an Illumina HiSeq 2500 platform, and 100 bp paired-end reads were generated.

### Read alignment and transcript assembly

Sequencing reads in FASTQ format were mapped to the human genome (hg19) using TopHat version 2.012^26^ with default parameters. The sample alignment data were then fed to a Cufflinks assembler, version 2.2.1^27^, to assemble the aligned reads into transcripts. All of the transcripts identified were merged into an integrated transcriptome using Cuffmerge software. The relative abundance of each transcript in the integrated transcriptome (of all samples) was re-estimated by Cuffquant in terms of the fragments per kilobase per million mapped reads (FPKM) and normalized with Cuffnorm. We filtered out the transcripts expressed at low levels and retained transcripts with FPKM < 0.01 in >75% of the samples.

**Table 1:** LincType genes in each subtype of breast cancer.

Subtype	LincType genes
Luminal A	BCLIN1, BCLIN2, BCLIN21, BCLIN22, BCLIN23, ASAH1, C6orf211, GLRB, KDM4B, PRKACB, RP11-265D17.2, SCCPDH, SCUBE2, SMOC2, TMEM26
Luminal B (HER2−)	BCLIN11, BCLIN12, BCLIN13, BCLIN14, BCLIN15, BCLIN16, BCLIN18, BCLIN19, BCLIN20, BCLIN21, BCLIN22, BCLIN23, C6orf211, GFRA1, KDM4B, PEG10, RP11-265D17.2, SCCPDH
Luminal B (HER2+)	BCLIN21, BCLIN22, KDM4B, BCLIN23, RP11-265D17.2, SCCPDH, C6orf211
HER2	BCLIN24, BCLIN25, BCLIN33, C1QL4, CDK12, PSAT1
Triple negative	BCLIN9, BCLIN10, BCLIN18, BCLIN19, BCLIN20, BCLIN24, BCLIN25, BCLIN26, BCLIN27, BCLIN28, BCLIN29, BCLIN30, BCLIN31, BCLIN32, PSAT1

### Identification of putative lincRNA transcripts in breast cancer samples

Novel lincRNAs among noncoding transcripts were identified in breast cancer samples according to the following method. First, transcripts that resided 2000 bp away from any known gene were selected based on RefSeq genes, Ensembl genes, GENCODE genes, and lincRNAs identified by Cabili et al. in 22 human tissues and cell lines. Only transcripts with a minimum length of 200 nt were retained. Finally, to obtain a reliable dataset of putative lincRNAs, we calculated the protein-coding capacity of novel transcripts using CPAT^28^ and iSeeRNA^29^ prediction software. Transcripts predicted by both programs as noncoding were designated verified, expressed, novel lincRNAs.

### Identification of differentially expressed transcripts

The Cuffdiff tool in the Cufflinks software package was employed to identify differentially expressed transcripts between each paired group, and all of the transcripts identified by this method were further analyzed. To verify these transcripts, we re-quantified the expression of the differentially expressed transcripts based on the large-scale RNA-Seq data deposited in TCGA.

### DNA methylation analysis

Based on the methylation data (batch A093) profile from the Infinium HumanMethylation450 BeadChip in TCGA, we identified CpG sites located 500–2000 bp away from a transcriptional start site for each differentially expressed known transcript. If the genes associated with these particular CpG sites were also expressed in all samples, the CpG sites and the associated genes were retained to calculate Pearson’s correlation coefficients, which were further used to evaluate the relationship between CpG methylation and gene expression.

### Construction of a coexpression network of differentially expressed transcripts

Pairwise expression correlations among the differentially expressed transcripts were determined using the same strategy employed for the characterization of GENCODE lncRNA catalog^12^. Briefly, a table for different classes of transcript pairs was constructed from the gene annotation table. These transcripts included known lincRNA-coding and novel lincRNA-coding pairs. For each pair of transcripts, Pearson’s correlation coefficients were calculated using FPKM expression estimates. Both trans (pairs consisting of transcripts located >1 Mb away from each another or located on different chromosomes) and cis (pairs consisting of transcripts located within a genomic window of 100 kb) correlations were calculated. To construct the coexpression network, we defined coding and noncoding transcripts as linked only when their associated Pearson’s correlation coefficient was significantly greater than 0.6 (*P* < 10^–5^). Two coding transcripts were considered linked if their Pearson’s correlation coefficient was significantly higher than 0.6 (*P* < 10^−5^) and if they were known to interact according to previously identified protein-protein interactions reported in the Bimolecular Interaction Network Database, the Biological General Repository for Interaction Datasets, the Database of Interacting Proteins, the Human Protein Reference Database, IntAct, the Molecular INTeraction database, the mammalian PPI database of the Munich Information Center on Protein Sequences, PDZBase (a PPI database for PDZ-domains) or Reactome. The network based on these links was visualized using Cytoscape^30^, and modules were identified using the ClusterOne plug-in embedded in Cytoscape.

### Clustering of transcripts based on FPKM

The hierarchical clustering of transcripts based on FPKM values across all samples was performed using GenePattern^31^. As the expression range of different transcripts varied between samples, row-center normalization was performed across all samples for each transcript. Then, the Euclidean distance was used as a distance measure for 2-way hierarchical clustering. The clustering results were visualized using HierarchicalClusteringViewer provided in GenePattern.

### Functional enrichment of coding transcripts and novel lincRNAs

DAVID Bioinformatics Resources^32^ was used to perform gene functional enrichment analysis of coding transcripts. GREAT software, which assigns biological meaning to a set of noncoding genomic regions by analyzing the annotations of neighboring genes^33^, was used to analyze the functions of differentially expressed novel lincRNAs. Functional enrichment analysis was performed using the default parameters provided by the indicated software.

### Identification of Linctype genes

To identify the transcripts with breast cancer-specific expression patterns, we classified all 33 samples into two groups, i.e., a breast cancer group and a normal control group, and used a feature selection approach (FilteredSubsetEval in WEKA). In this manner, we identified 79 transcripts with breast cancer-specific expression patterns. For classifying all 33 samples into six groups, namely, five breast cancer subtypes and a normal control, the same feature selection approach was applied to the 79 transcripts to identify transcripts with subtype-specific expression patterns. In this manner, we identified 44 group-specific transcripts. Moreover, for each breast cancer subtype, specifically expressed transcripts [4 for luminal B (HER2−), 1 for luminal B (HER2+), 2 for triple negative, 2 for luminal A, and 1 for HER2] were selected. In addition, we selected six transcripts with markedly low expression levels and three transcripts with markedly high expression levels in all of the breast cancer samples. In total, we obtained 60 transcripts and designated them Linctype genes (Table 1).

### Validation of Linctype genes using qPCR

Total RNA was extracted from 250 freshly frozen samples using TRIzol reagent (Invitrogen, Carlsbad, CA) according to the manufacturer’s instructions. Total RNA (2 µg) was reverse transcribed using Transcriptor First Strand cDNA Synthesis Kit (Roche, Vilvoorde, Brussels, Belgium). The relative levels of genes and control GAPDH were determined by qRT-PCR using a Roche LightCycler® 480 (LC480) Real-Time PCR Platform. Primer and siRNA sequences are shown in Additional file 1: Table S1. The level of each transcript relative to control GAPDH transcript was determined by the 2^−ΔCT^ method^34^.

### Cell culture, treatments, and proliferation assays

Normal mammary cells(MCF-10A), breast cancer cell lines (MDA-MB-231, MDA-MB-453, MDA-MB-468, Hs-578T, MCF-7, UACC-812,SKBR3,T47D, and BT-549) and the human embryonic kidney (HEK) 293T cell line were obtained from the Chinese Academy of Sciences Cell Bank and Cellbio (China) and were cultured according to the suppliers’ instructions.

Cell proliferation assays were performed using Cell Counting Kit-8 (CCK-8) according to the manufacturer’s instructions (Beyotime, Shanghai, China). Briefly, 2 × 10^3^ cells were seeded in a 96-well plate. Cell proliferation was assessed at 24, 48, and 72 h. After the addition of 20 μL of WST-1 reagents per well, the cultures were incubated for 2 h, and the absorbance was measured at 450 nm using a microplate reader (BioTek, VT, USA).

### Cell migration and invasion assays

Migration and invasion assays were performed in 24‑well cell culture inserts (Corning) fitted with a PET membrane (8-μm pore size). The inserts for invasion assays were coated with 30 μL Matrigel matrix at 37 °C for 1 h. Transfected cells were plated in medium without serum in the top chamber of a transwell. The bottom chamber contained 600 μL RPMI 1640 medium with 10% FBS. After incubating for 24 h at 37 °C, the cells that had migrated to the lower surface of the membrane were fixed with 4% methanol, stained with crystal violet and photographed under a microscope. Cell numbers were counted under a light microscope at 200 × magnification.

### TUNEL assays and flow cytometry

Apoptosis-induced DNA fragmentation was examined with the transferase-mediated deoxyuridine triphosphate (dUTP)-digoxigenin nick end-labeling (TUNEL) assay, and the experiment was performed as previously described^35^. Briefly, cells were plated in 24-well flat-bottom plates and fixed in 4% (w/v) paraformaldehyde at 4 °C for 25 min, and TUNEL and DAPI staining were performed for 10 min according to the manufacturer’s instructions. The numbers of TUNEL-positive cells were evaluated with a fluorescence microscope (Olympus), and the ratio of apoptotic cells was determined with Image-Pro Plus software. For cell cycle analysis, a CycleTEST™ PLUS DNA Reagent Kit (Catalog Number 340242, BD) was used, and the experiment was performed as previously described^35^.

### Subcellular fractionation

Nuclear/cytoplasmic extract isolation was carried out using NE-PER^TM^ Nuclear and Cytoplasmic Extraction Reagents (Catalog Number 78835, Thermo Fisher, USA) according to the manufacturer’s recommendation. Cytoplasmic and nuclear fractions were split for RNA extraction. GAPDH and U6 were used as markers of cytoplasmic and nuclear fractions by qRT-PCR.

### Luciferase reporter assay

For luciferase reporter assays, HEK-293T cells (5 × 10^4^) grown to 80% confluence in 24-well plates were cotransfected with miR-125b mimics, miR-125b inhibitors, or control oligonucleotides with reporter plasmids using Lipofectamine 2000. After 48 h of transfection, the cells were lysed in passive lysing buffer and then analyzed for firefly and Renilla luciferase activities using the commercial Dual-Luciferase Reporter Assay System (E1910, Promega) according to the manufacturer’s instructions. Cells transfected with the control vector were used to check transfection efficiency, and Renilla luciferase was used as a normalization control.

### Western blot analysis

Cells were lysed in lysis buffer. Protein samples were separated by SDS-PAGE and transferred into a 0.22 μm polyvinylidene fluoride (PVDF) membrane. Proteins were probed with antibodies against ERBB2 (TA321952, OriGene), Bcl-2 (#2876S, CST,) and Bax (#2774S, CST). The bound antibodies were detected using an ECL Western Blotting Detection system. Internal reference of protein loading was assessed using Tubulin (wl01783, Wanleibio). Each experiment was conducted three times.

### Animal experiments

All experimental procedures involving animals were performed in accordance with animal protocols approved by the Animal Care and Use Committee of Harbin Medical University. Four-week-old female BALB/c nude mice were obtained from Shanghai Laboratory Animal Center, CAS, and housed in a pathogen-free and temperature-controlled environment. Approximately 3–8 × 10^6^ cells suspended in 0.2 ml of 25% phenol red-free Matrigel (Catalog Number 356234, Corning) were subcutaneously inoculated into the right mammary fat pad. The size of the tumors was measured with a caliper every 3 days and calculated as length × width^2^ × 1/2. Bioluminescent imaging was performed using a highly sensitive, cooled charge-coupled device camera mounted in a light-tight specimen box (IVIS 200, Xenogen), with protocols similar to those described previously^36^.

### Bisulfite sequencing

Genomic DNA was isolated from UACC-812 cells using an AxyPrep^TM^ Multisource Genomic DNA Miniprep Kit (Axygen Scientific, San Francisco, USA) according to the manufacturer’s instructions. DNA was bisulfite converted using the EZ DNA Methylation-Gold kit (Zymo Research, Orange, USA) and amplified using MegaMix Gold 2 × Mastermix and validated primer pairs. PCR products were cloned into the pTG19-T (Lot: GV6021) vector and sequenced at a depth of ~500× according to previously described methods^37^.

### Statistical analyses

Differences between each group in vitro were analyzed using Student’s *t*-tests. Spearman’s correlation coefficients were calculated for correlation analysis. Overall survival (OS) and disease-free survival (DFS) were calculated as the time from surgery until the occurrence of death or relapse, respectively. The expression of BCLIN25 was dichotomized using a study-specific median expression as the cut-off to define “high value” at or above the median versus “low value” below the median. All in vitro experiments were performed in triplicate. All statistical tests were two-sided, and *P* < 0.05 was regarded as statistically significant.

## Results

### Identification landscape of novel lincRNAs in breast cancer

**Fig. 1:**
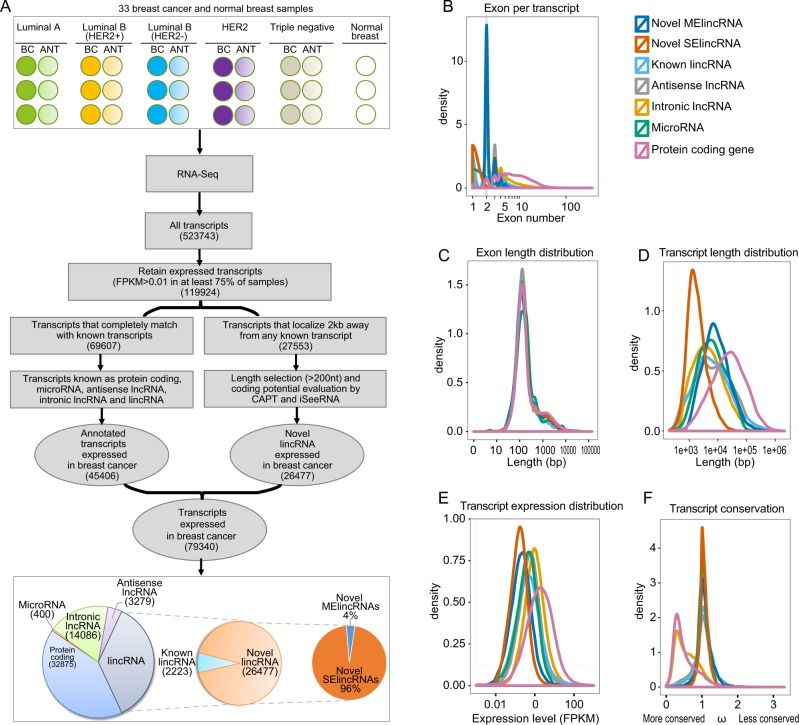
Identification landscape of novel lincRNAs in breast cancer. **a** Schematic overview of the methodology employed in this study. Graphical representation of the bioinformatics filters used to merge individual transcriptome libraries into a single consensus transcriptome. A merged consensus transcriptome was generated by compiling individual transcriptome libraries and discarding low-confidence transcripts. The remaining transcripts were categorized as annotated protein-coding, noncoding or unannotated transcripts based on their similarity to genes from an aggregated set of known annotated genes. After filtering for length and coding potential, the remaining unannotated transcripts were considered novel lincRNAs expressed in breast cancer. **b** Numbers of exons per transcript for novel SElincRNAs, MElincRNAs, known lincRNAs, lincRNAs, microRNAs, and protein-coding genes are represented by different colored lines. **c** Exon length distribution of different transcript types. **d** Transcript length distributions of different transcript types. **e** Transcript expression distributions of different transcript types. **f** Level of conservation between different transcript types.

To annotate previously unreported lincRNAs associated with breast cancer, we performed paired-end whole transcriptome sequencing (RNA-Seq) using RNA for 33 samples from 11 groups encompassing tissues representing five breast cancer subtypes [luminal A, luminal B (HER2+), luminal B (HER2−), HER2, and triple negative], adjacent noncancerous breast tissues, and normal breast tissues according to St Gallen International Expert Consensus^8^ (Additional file 1: Table S2). A total of 1.63 billion short reads were generated, and 1.51 billion mapped reads with a median of 45.58 million reads per sample were obtained (Fig. 1a and Additional file 1: Table S3). The majority of transcripts (58.0%) corresponded to annotated protein-coding genes (27.4%) and noncoding RNAs (30.6%), but a substantial percentage (23.0%) of transcripts were located 2 kb away from any known transcript and were not annotated (Additional file 1: Table S3). Finally, we obtained reliably expressed lincRNAs, and these novel lincRNAs were combined with 45,406 annotated transcripts to generate a consensus, composite transcriptome for further analysis (Additional file 1: Table S3).

Further analysis revealed that most (96%) of the novel lincRNAs identified represented single-exon lincRNAs (SElincRNAs), while only 4% represented multiple-exon lincRNAs (MElincRNAs) (Fig. 1a). The latter group of lincRNAs had fewer exons than protein-coding genes (2.33 versus 9.50 on average), but their exon lengths were similar to those of protein-coding genes (Fig. 1b, c). The novel MElincRNAs transcripts exhibited comparable lengths to known noncoding RNAs (Fig. 1d). Full-length novel MElincRNAs (median length of 7974 nt) were longer than SElincRNAs (median length of 1673 nt) but generally shorter than protein-coding transcripts (median length of 23,425 nt). Additionally, the expression levels of novel lincRNAs were lower than those of protein-coding transcripts (Fig. 1e). The conservation of novel lincRNAs in breast cancer was further examined using multi-way genomic alignments across 29 placental mammals. Both novel SElinRNAs and MElincRNAs displayed similar conservation distributions with known lincRNAs. In addition, both novel and known lincRNAs exhibited less conservation than protein-coding genes, consistent with data from previous studies^38,39^ (Fig. 1f).

### Expression diversity of lincRNAs across breast cancer subtypes

**Fig. 2:**
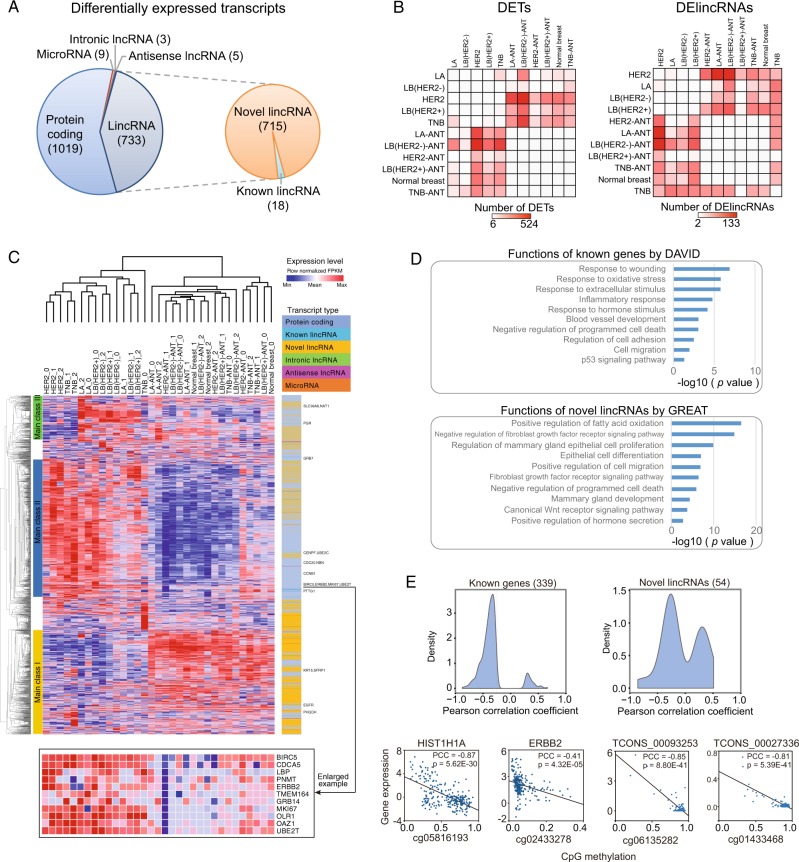
Differentially expressed transcripts in different groups of breast cancer tissue samples. **a** Compilation of differentially expressed transcripts. **b** Number of transcripts and novel lincRNAs differentially expressed between the two groups. **c** Two-way hierarchical clustering of differentially expressed transcripts and breast cancer tissue samples. The top panel displays the sample information and clustering results, and the left panel displays the three major classes of genes. The right panel lists the type of transcript associated with each gene and with established breast cancer genes. The bottom panel portrays the expression levels for key breast cancer genes, including ERBB2 and MKI67. **d** Functional enrichment analysis and KEGG pathway analysis of differentially expressed coding genes using DAVID, and enriched functions of differentially expressed novel lincRNAs according to GREAT. **e** Promoter DNA methylation regulates the expression of transcripts differentially expressed in breast cancer tissues. The distribution of Pearson’s correlation coefficients between known genes and novel lincRNAs. Examples are presented at the bottom.

To further characterize the lincRNA expression profile obtained from our RNA-Seq analysis, we identified the transcripts that were differentially expressed between groups. Only 2.3% (1769) of the transcripts were differentially expressed across different breast tissues. The differentially expressed transcripts included 1019 protein-coding transcripts, 9 microRNAs, 8 lincRNAs, 18 known lincRNAs, and 715 novel lincRNAs (Fig. 2a). Most of the differentially expressed transcripts were identified based on comparisons between breast cancer tissues and either adjacent noncancerous tissues or normal breast tissues (Fig. 2b), suggesting that different types of breast cancer might exhibit similar lincRNA expression aberrations in most cases. These results were also verified using 2-way hierarchical clustering (Fig. 2c). The transcripts were further grouped into different classes. Class-I transcripts were significantly downregulated in breast cancer, while Class-II transcripts were significantly upregulated in breast cancer. Additional analysis revealed that most Class-II transcripts were expressed from protein-coding genes, including the well-characterized breast cancer genes ERBB2 and MKI67, while most Class-I transcripts were novel lincRNAs whose function and significance in breast cancer had not been previously characterized. In addition, some transcripts revealed the existence of subtype-specific expression patterns. For example, Class-III transcripts were highly expressed in luminal breast cancer.

Next, the functional roles of protein-coding transcripts and novel lincRNA transcripts that were differentially expressed in breast cancer were investigated. The results revealed that the differentially expressed coding genes were significantly enriched for those involved in breast cancer-associated biological progression, such as wound healing, biological responses to hormone stimulation, blood vessel development and programmed cell death inhibition (Fig. 2d). Moreover, 671 of the differentially expressed novel lincRNAs were located near 566 known coding genes (Additional file 2: Fig. S1a, b), and among these lincRNAs, 501 were located near 283 protein-coding genes that were differentially expressed in breast cancer. Consistent with this finding, 50 of the 283 protein-coding genes, including the previously characterized ESR1, AR and MKI67, were identified as differentially expressed in this study (Additional file 2: Fig. S1c). The functions of the coding genes located near the differentially expressed novel lincRNAs were associated with tumorigenesis, including fatty acid oxidation (IRS2, PPARA), cell proliferation, epithelial cell differentiation, programmed cell death and mammary gland development (Fig. 2d).

The cause of the differential expression pattern was further explored. For protein-coding genes, 27% (339) of the 1255 CpG sites were significantly (*P* < 0.01) associated with transcript expression levels (Fig. 2e; Additional file 1: Table S4), and the majority of these CpG modifications were associated with the negative regulation of corresponding genes. For example, the most significant (*P* = 5.62E−30) association was observed between the HIST1H1A gene and the corresponding CpG site cg05816193 (Fig. 2e). The expression of ERBB2 also demonstrated a significant inverse correlation with the methylation level at the cg02433278 CpG site in its promoter region (Fig. 2e). For novel lincRNAs, DNA methylation patterns at the 54 CpG sites exhibited a significant correlation (*P* < 0.01) with the expression levels of the respective novel lincRNA. For example, two novel lincRNAs, TCONS_00093253 and TCONS_00027336, were inhibited by CpG methylation. Collectively, these data indicated that both protein-coding genes and novel lincRNAs differentially expressed in breast cancer tissues may be regulated, in part, by DNA methylation.

### Correlation between lincRNA and protein-coding transcripts

**Fig. 3:**
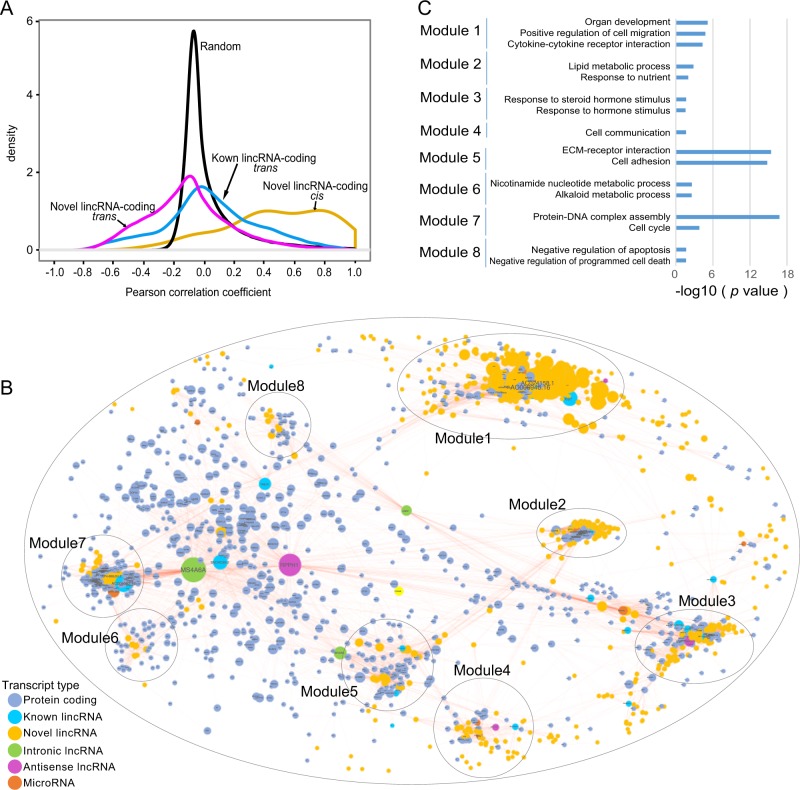
Novel lincRNA genes are coexpressed with protein-coding genes associated with cell migration, hormone stimulus responses, and cell adhesion. **a** Density histograms of pairwise Pearson’s expression correlations between lincRNAs and protein-coding genes in trans or cis. **b** Coexpression network of differentially expressed transcripts. Nodes represent the differentially expressed transcripts, and lines represent the significant coexpression transcript pairs. **c** Significantly enriched biological functions associated with the differentially expressed coding genes of different modules in (**b)**.

The coexpression patterns of novel lincRNAs and protein-coding transcripts were next examined. Both known and novel lincRNAs were more closely associated with their corresponding protein-coding genes compared with a random control set in which the expression of protein-coding genes was randomly shuffled (Fig. 3a). Notably, the expression of cis pair components was more likely to be directly correlated (41.4% of the pairs exhibited a Pearson’s correlation coefficient >0.6 versus only 2.8% in the control set based on the Chi-square test, *P* *=* 5.9 × 10^−33^), whereas the expression of trans pair components was more likely to be inversely correlated (3.0% of the pairs had a Pearson’s correlation coefficient < −0.6, versus 0% in the control set). We subsequently constructed a network based on the coexpression of novel lincRNAs and known genes and further divided the network into five submodules (Fig. 3b). Functional enrichment analysis of the coding genes in each of the 8 main modules suggested that coexpressed genes shared similar functions (Fig. 3c, Additional file 1: Table S5). Module 1 was enriched for genes associated with organogenesis and cell migration, and Module-3 was enriched for genes associated with hormone stimulus responses. Module 5 exhibited a significant enrichment for genes associated with cell adhesion, and Module 7 was enriched in genes associated with cell cycle regulation.

### LincRNA-based classification of breast cancer subtypes

**Fig. 4:**
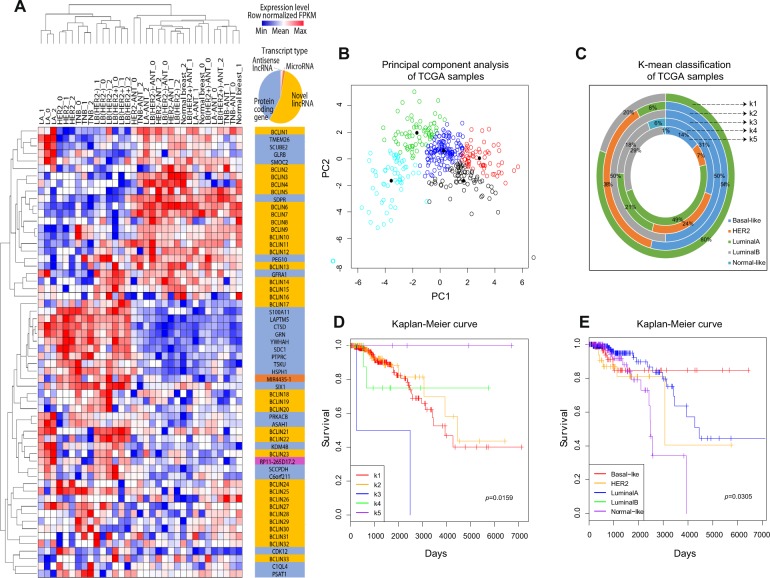
LincRNA-based classification of breast cancer subtypes. **a** Unique set (Linctype) of genes including novel lincRNAs for breast cancer subtype classification. **b** Principal component analysis of TCGA samples. The first two principal components were used to map the scatter plot of samples. Different colored nodes represent different classes identified by principal component analysis. **c** Classification of TCGA samples by K-mean clustering of gene expression in the Linctype. The composition of subtypes following PAM50 analysis in each cluster (k1, k2, k3, k4, and k5) is shown with each circle. d Kaplan–Meier curves of five clusters identified using genes in the Linctype. e Kaplan–Meier curves of five subtypes identified using PAM50.

WEKA was used to analyze the expression profiles of differentially expressed genes and MS-lincRNAs. In this process, we established a novel breast cancer subtype classification system containing 60 genes with subtype-specific expression patterns, of which 33 were MS-lincRNAs (Fig. 4a; Table 1). We termed this lincRNA-associated subtype classification system Linctype (Additional file 2: Fig. S2; Additional file 1: Tables S1 and S6). In the Linctype, 17 genes were associated with the classic breast cancer subtyping method PAM50^40^ (Additional file 2: Fig. S3). Hierarchical clustering revealed that Linctype genes can be used not only to distinguish breast cancer tissues from normal tissues but also to correctly classify these tissues into 5 distinct subtypes (Fig. 4a). Furthermore, Linctype genes could reclassify the subtypes of TCGA samples by principal component analysis and K-means clustering, respectively. Principal component analysis and K-means clustering suggested that five breast cancer groups were present in samples from TCGA (Fig. 4b, c). Survival analysis indicated that these sample groups showed significant differences in OS rates (Fig. 4d), and the difference was more significant than that based on PAM50 (Fig. 4e). Therefore, the novel Linctype genes might serve as a useful complement to the current breast cancer subtyping system.

### Validation of MS-lincRNAs

**Fig. 5:**
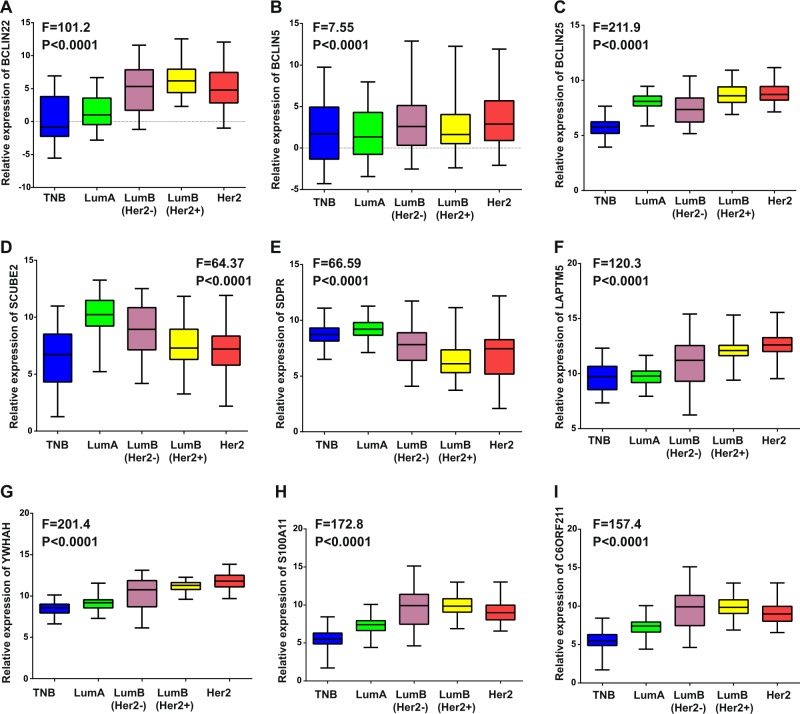
Validation of MS-lincRNAs via qRT-PCR in 250 breast cancer tissues in the HMUCC cohort. **a** BCLIN22 was highly expressed in luminal B (HER2+) breast cancer. **b**, **c** BCLIN5 and BCLIN25 were highly expressed in HER2 breast cancer. **d**, **e** SCUBE2 and SDPR were highly expressed in luminal A breast cancer. **f**, **g** LAPTM5 and YWHAH were highly expressed in HER2 breast cancer. h&i S100A11 and C6ORF211 in luminal B breast cancer.

The identified MS-lincRNAs were next validated using 250 breast cancer tissues in our cohort. The MS-lincRNAs BCLIN22 and were highly expressed in luminal B (HER2+) breast cancer (Fig. 5a). Moreover, MS-lincRNA BCLIN5 and BCLIN25 were highly expressed in HER2 breast cancer (Fig. 5b, c). For protein-coding genes, SCUBE2 and SDPR were highly expressed in the luminal A subtype, LAPTM5 and YWHAH in the HER2 subtype, and S100A11 and C6ORF211 in the luminal B subtype of breast cancer (Fig. 5d–i). Other MS-lincRNAs and subtype-specific coding genes were next evaluated in TCGA and GEO datasets. BCLIN27, BCLIN29, and BCLIN30 were identified as MS-lincRNAs in basal-like breast cancer, BCLIN21 in luminal A breast cancer, BCLIN16 in luminal B breast cancer and BCLIN25 in HER2 breast cancer (Additional file 2: Fig. S4a–f). Moreover, SCUBE2, TMEM26, and SMOC2 were validated as subtype-specific coding genes in luminal A breast cancer, CDK12 and SDC1 in HER2 breast cancer and PSAT1 in triple negative breast cancer in TCGA and GEO datasets (Additional file 1: Fig. S4g–l and Fig. S5).

### MS-lincRNA BCLIN25 contributes tumorigenesis and progression in HER2 subtype breast cancer

**Fig. 6:**
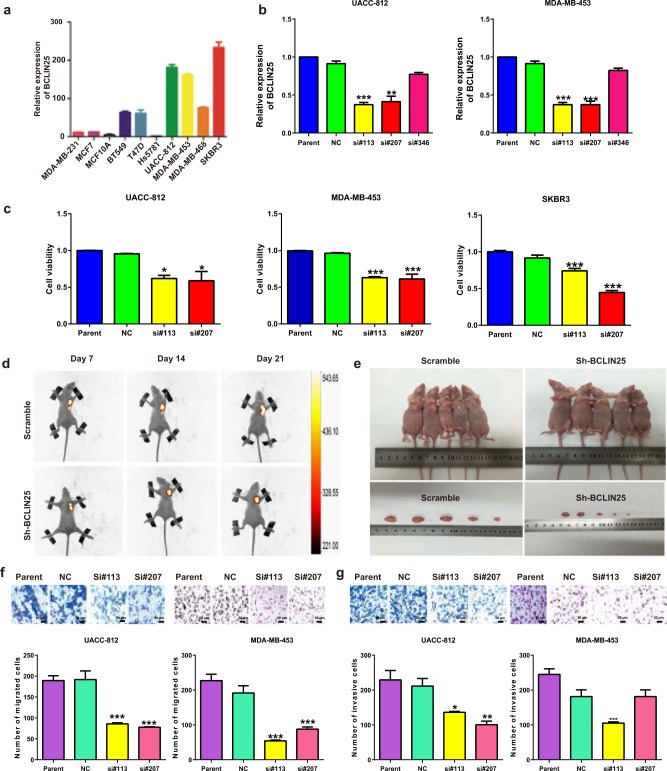
MS-lincRNA BCLIN25 contributes tumorigenesis and progression in HER2 breast cancer cells. **a** Expression of BCLIN25 in breast cancer cell lines. **b** Knockdown efficiency of siRNAs targeting BCLIN25 in UACC812 and MDA-MB-453 cells. **c** Viability was inhibited in the BCLIN25 knockdown group compared with that in the control group, as shown by CCK-8 assays in UACC812, SKBR3, and MDA-MB-453 cells. **d** Tumors with or without BCLIN25 knockdown in nude mice by bioluminescence imaging. **e** Tumor samples with or without BCLIN25 knockdown in nude mice. **f** Cell migration was decreased in the BCLIN25 knockdown group compared with that the control group, as shown by transwell assays in UACC-812 and MDA-MB-453 cells. **g** Cell invasion was decreased in the BCLIN25 knockdown group compared with that in the control group, as shown by transwell assays in UACC-812 and MDA-MB-453 cells. *P *<* 0.05; **P < 0.01; ***P < 0.001. Data represent at least three independent experiments.

**Fig. 7:**
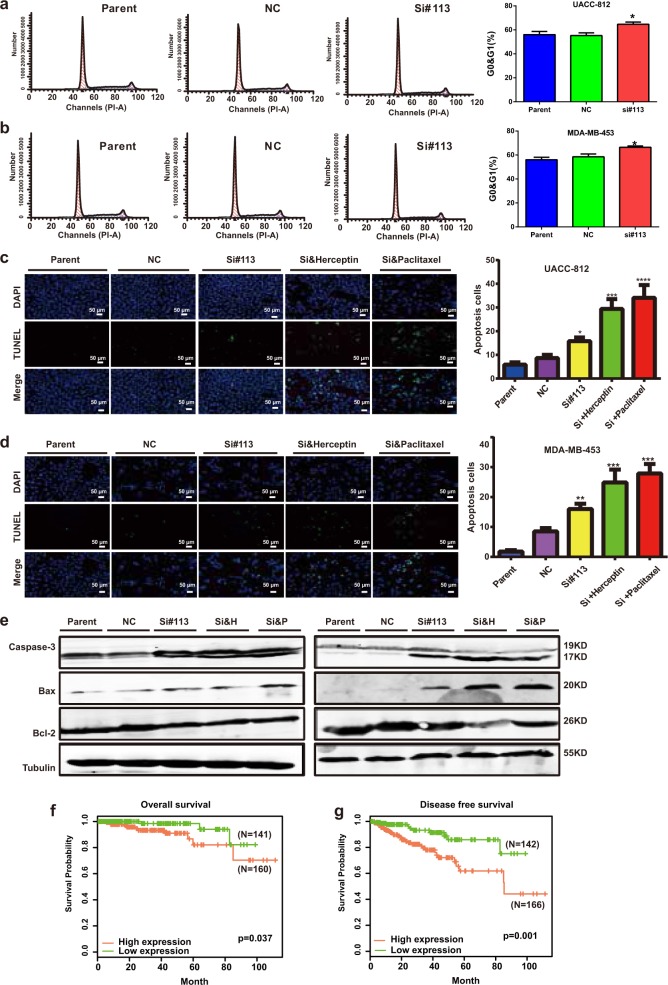
MS-lincRNA BCLIN25 knockdown plays synergistic roles with targeted therapy or chemotherapy in breast cancer cells. **a**, **b** The G0/G1 cell ratio was increased in the BCLIN25 knockdown group compared with that in the control group, as shown by flow cytometric examinations in UACC-812 and MDA-MB-453 cells. **c**, **d** Cell apoptosis was increased in the BCLIN25 knockdown group with or without Herceptin or paclitaxel compared with that in the control group, as shown by TUNEL assays in UACC-812 and MDA-MB-453 cells. **e** Caspase-3, Bax, and Bcl-2 protein expression in the BCLIN25 knockdown group and the control group of UACC812 and MDA-MB-453 cell lines with or without Herceptin/paclitaxel treatment in UACC-812 (left) and MDA-MB-453 cells (right). **f**, **g** High expression of BCLIN25 leads to worse OS and DFS in breast cancer patients administered paclitaxel-containing chemotherapy regimens based on TCGA data. *P *<* 0.05; **P < 0.01; ***P < 0.001; and ****P < 0.0001. Data represent at least three independent experiments.

Because BCLIN25 was identified as a HER2 subtype-specific lincRNA in breast cancer, its potential biological function was subsequently investigated. The expression of BCLIN25 was examined in breast cancer cell lines. BCLIN25 expression was higher in Her2 breast cancer cell lines than in other cell lines (Fig. 6a). Next, siRNAs targeting BCLIN25 were designed, and the knockdown efficiency was examined in UACC812 and MDA-MB-453 cell lines (Fig. 6b). Compared with that in the control group, cell viability was inhibited in the BCLIN25 knockdown group, as shown by CCK-8 assays in UACC812, SKBR3 and MDA-MB-453 cell lines (Fig. 6c). Moreover, based on in vivo assays, tumors in the BCLIN25 knockdown groups grew more slowly than those in the scrambled group (Fig. 6d, e). Cell migration and invasion were also decreased in the BCLIN25 knockdown group compared with those in the control group, as shown by transwell assays in the above three cell lines (Fig. 6f, g). The G0/G1 cell ratio was also increased in the BCLIN25 knockdown group compared with that in the control group (Fig. 7a, b). These results were also confirmed by TUNEL assays in these cell lines with or without Herceptin or paclitaxel treatment (Fig. 7c, d). Furthermore, the levels of apoptosis markers such as Caspase-3 and Bax were increased, while that of Bcl-2 was decreased in the BCLIN25 knockdown group (Fig. 7e). The result was even more significant when BCLIN25 knockdown was combined with Herceptin treatment (Fig. 7e). Moreover, high expression of BCLIN25 was also related to unfavorable OS and DFS in breast cancer patients receiving paclitaxel-containing chemotherapy regimens in TCGA datasets (Fig. 7f, g).

### BCLIN25 is upregulated by epigenetic activation and promotes ERBB2 expression by suppressing miR-125b expression

**Fig. 8:**
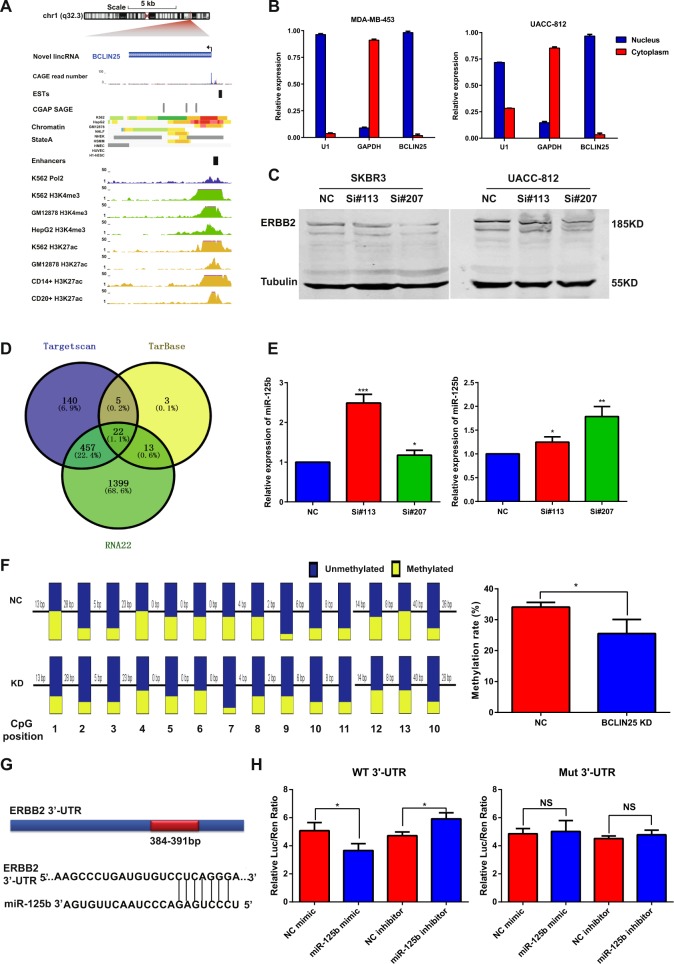
BCLIN25 is upregulated by epigenetic activation and promotes ERBB2 expression by suppressing miR-125b expression. **a** Activated transcription start site together with H3K4m3, H3K27ac and Pol2 enrichment were observed in the BCLIN25 locus based on the analysis of ChIP-seq data from ENCODE. **b** Localization of BCLIN25 in MDA-MB-453 and UACC-812 cells, as shown by fractionated nuclear and cytoplasmic RNA assays. **c** ERBB2 was decreased in the BCLIN25 knockdown group compared with that in the control group of SKBR3 and UACC-812 cells. **d** Prediction of potential microRNAs that bind the 3’-UTR of ERBB2, as shown by the integrated analysis of TargetScan (http://targetscan.org/), TarBase (http://diana.imis.athena-innovation.gr/DianaTools/index.php?r=tarbase/index) and RNA22 (https://cm.jefferson.edu/rna22/) databases. **e** miR-125b was upregulated in the BCLIN25 knockdown group of UACC-812 (left) and MDA-MB-453 cells (right). **f** Promoter CpG methylation of miR-125b was decreased in the BCLIN25 knockdown group compared with that in the control group of UACC-812 cells, as shown by the bisulfite sequencing. **g** Potential binding site of miR-125b in the 3′-UTR of ERBB2. **h** Luciferase reporter assays after the cotransfection of miR-125b precursor with wild-type reporter or reporters containing mutations at putative miR-125b-binding sites. *P *<* 0.05; **P < 0.01; ***P < 0.001. Data represent at least three independent experiments.

By the analysis of ChIP-seq data from ENCODE, an activated transcription start site was observed at the BCLIN25 locus (Fig. 8a). Moreover, epigenetic activation markers H3K4m3 and H3K27ac accompanied by Pol2 were also enriched at the promoter of BCLIN25 (Fig. 8a, Supplementary Fig. S6). These results indicated that the transcriptional activation of BCLIN25 was partly mediated by an epigenetic mechanism. BCLIN25 is mainly located in the nucleus, as shown by fractionated nuclear and cytoplasmic RNA assays in MDA-MB-453 and UACC-812 cell lines, suggesting that BCLIN25 regulates its target genes either by transcriptional modulation or by chromatin modification (Fig. 8b).

Because BCLIN25 is a HER2 subtype-specific-lincRNA in breast cancer, we wondered whether ERBB2 is a downstream target of BCLIN25. Interestingly, compared with that in the control group, ERBB2 expression was decreased in the BCLIN25 knockdown group of SKBR3 and UACC-812 cells (Fig. 8c). MicroRNAs have been reported to be important regulators of gene expression. Because of its nuclear location, BCLIN25 may regulate ERBB2 expression via epigenetic modification of microRNAs in the nucleus. Thus, TargetScan, TarBase and RNA22 databases were used to predict the potential microRNAs that bind the 3’-UTR of ERBB2 (Fig. 8d; Additional file 1: Table S7). Based on the data, miR-125b and miR-133, owing to their substantial binding potential to ERBB2 3’-UTR, were selected for subsequent validation. miR-125b was upregulated in the BCLIN25 knockdown group, while miR-133 was not (Fig. 8e, f; Additional file 2: Fig. S7a). Furthermore, promoter CpG methylation of miR-125b was decreased in the BCLIN25 knockdown group compared with that in the control group (Fig. 8g). Thus, BCLIN25 may increase miR-125b promoter CpG methylation, leading to the downregulation of miR-125b.

Moreover, the introduction of miR-125b mimics decreased the expression of ERBB2 mRNA and protein levels (Additional file 2: Fig. S7b, c). To test whether miR-125b directly binds to the 3′-UTR of ERBB2 mRNA, we predicted the potential binding region via bioinformatics analysis and constructed ERBB2 3′-UTR reporters containing putative miR-125b-binding sites and corresponding mutated versions downstream of a luciferase reporter gene (Fig. 8h). Cotransfection of miR-125b precursor with wild-type reporter constructs greatly decreased luciferase activities, while cotransfection with reporters containing mutations at putative miR-125b-binding sites did not affect luciferase activities (Fig. 8i). These results confirmed that ERBB2 is a direct target of miR-125b. Thus, ERBB2 is regulated by the BCLIN25-miR-125b-ERBB2 axis, and BCLIN25 is a bona fide MS-lincRNA in HER2 breast cancer.

## Discussion

LincRNAs play important roles in regulating protein functions associated with tumorigenesis and have been used as biomarkers for diagnosis, therapy selection and prognostic prediction in human cancer. Therefore, it is critical to further characterize the landscape of lincRNAs and elucidate their potential roles in human cancer. In this study, the transcriptional landscape of novel lincRNAs associated with breast cancer subtypes was determined using RNA transcriptome profiling in five major subtypes of human breast cancer. To this end, previously unknown lincRNAs representing novel lincRNAs associated with breast cancer were systematically identified through the analysis of transcriptomes across different breast cancer subtypes. Further stratification of the differentially expressed lincRNAs enabled the establishment of a novel breast cancer subtype classification system, termed Linctype, including MS-lincRNAs and coding genes. The Linctype could not only distinguish breast cancer tissues from normal tissues but also classify breast cancer tissues into five distinct molecular subtypes associated with significant prognostic differences. Independent qPCR validation in our cohort and analysis of data from TCGA and GEO confirmed the differential expression pattern of these MS-lincRNAs in a large number of breast cancer tissues. Finally, as a HER2 subtype-specific lincRNA, BCLIN25 was selected, and its biological function and contribution to tumorigenesis were investigated in vitro and in vivo. BCLIN25 was shown to increase the expression of ERBB2 by enhancing CpG methylation of the miR-125b promoter, leading to the downregulation of miR-125b. In turn, ERBB2 mRNA degradation was found to be abolished due to decreased binding of miR-125b to the 3’-UTR of ERBB2. Thus, the BCLIN25-miR-125b-ERBB2 axis was identified to upregulate ERBB2 expression, thereby indicating BCLIN25 as a bona fide MS-lincRNA for HER2 breast cancer. Our results indicate that lincRNAs may be involved in tumorigenesis and could aid in breast cancer molecular classification.

A major challenge in studying novel lincRNAs is determining the basic characteristics and potential biological roles of the lincRNAs. Although some previous studies have solely evaluated MElincRNAs, SElincRNAs also play important roles. For example, NEAT1, a 22.7-kb SElincRNA, is required for the formation of nuclear paraspeckles^41^. Here, we report that SElincRNAs are conserved in similar species and participate in regulating nearby protein-coding genes. Although the function of most lincRNAs remains unknown, well-established oncogenes have been found to be associated with lincRNAs, such as lincRNA-p21^42^, HOTAIR^43^, MALAT-1^44^, and PCAT-1^16^, indicating that lincRNAs may be essential factors in cancer pathogenesis. In this study, MS-lincRNA BCLIN25, which serves as a HER2 subtype-specific lincRNA, was found to contribute to tumorigenesis by upregulating ERBB2 expression via epigenetic modification and RNA–RNA interactions in breast cancer. LincRNAs have many different functions and are also associated with structural molecules^45^. Currently, lincRNAs are considered to act as cis-activators and to exhibit enhancer-like functions based primarily on their proximity to protein-coding genes^9,46,47^. Tomita et al. recently identified a cluster of noncoding RNAs that can activate the ESR1 locus during breast cancer development^48^. Consistent with this finding, we found that most of the differentially expressed novel lincRNAs that we identified are likely to function as cis-activators of nearby protein-coding genes involved in breast cancer initiation and progression.

In the clinic, although intrinsic breast cancer subtypes defined by pathologic classification of ER, PR, HER2, and Ki-67 status facilitate the development of precise treatment approaches, the use of these methods remains far from perfect due to subjectivity among pathologists in interpreting test results, diversity of antibody quality, and lack of standardize immunohistochemical protocols among hospitals. To solve this problem, the establishment of a novel subtype classification system that could complement the current subtyping method is needed. Here, we reported an optimal subset of lincRNAs that can be used for the molecular subtyping of breast cancer tissues (termed Linctype). The Linctype comprises 60 transcripts, 33 of which are novel lincRNAs. To ameliorate the limitation of the small number of samples used in RNA-Seq profiling, we refined and validated the Linctype from a large number of samples from TCGA by the process of elimination and computational analysis. To date, this study represents the first integrated analysis of novel lincRNA expression and protein-coding gene expression for breast cancer subtyping. The capability of the Linctype to classify breast cancer tissues into different subtypes suggested that novel lincRNAs have a potential role in breast cancer subtypes. Our data also indicate that the Linctype may complement current subtyping methods. Thus, a novel breast cancer subtyping system characterized by lincRNA expression analysis combined with immunohistochemical detection may be more specific and accurate than a system using protein-coding genes alone. The sensitivity and specificity of the novel subtyping system combined with immunohistochemical detection will be explored in detail in multiple cohorts with large populations in future studies.

In conclusion, an extensive landscape of global novel lincRNA expression patterns revealed new relationships between novel lincRNAs and breast cancer subtypes. These findings may provide important insights into better ways of classifying molecular subtypes of breast cancer and facilitate the development of more precisely tailored treatment approaches.

## Supplementary information

Supplementary Figure 1

Supplementary Figure 2

Supplementary Figure 3

Supplementary Figure 4

Supplementary Figure 5

Supplementary Figure 6

Supplementary Figure 7

Supplementary file legends

Supplementary Table 1

Supplementary Table 2

Supplementary Table 3

Supplementary Table 4

Supplementary Table 5

Supplementary Table 6

Supplementary Table 7

LANGUAGE EDITING CERTIFICATE

Reporting Checklist

DECLARATION OF CONTRIBUTIONS TO ARTICLE

## Data Availability

Raw sequencing and processed RNA-Seq data from this study have been deposited into the NCBI GEO database under accession number GSE71651.
