# Supplementary figures and images for: The MS-lincRNA landscape reveals a novel lincRNA BCLIN25 that contributes to tumorigenesis by upregulating ERBB2 expression via epigenetic modification and RNA–RNA interactions in breast cancer
Source: Cell Death Dis. 2019 Dec 4;10(12):920. doi: 10.1038/s41419-019-2137-5 (PMC6892920; doi:10.1038/s41419-019-2137-5)

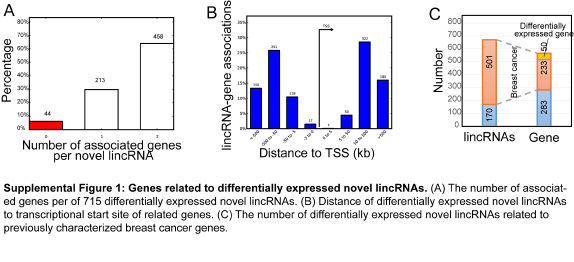

Supplement: Supplementary file 1 — Supplementary Figure 1 [file 41419_2019_2137_MOESM1_ESM.png]

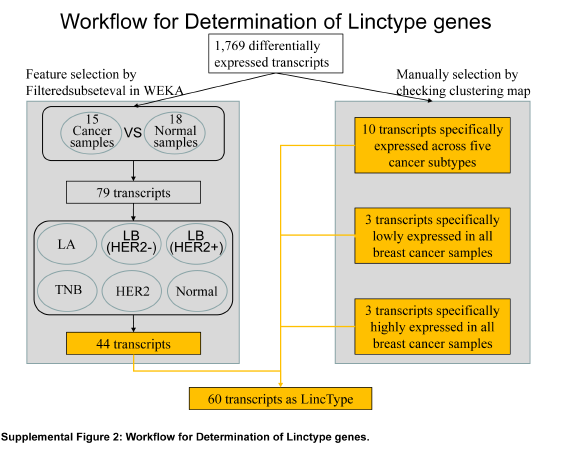

Supplement: Supplementary file 2 — Supplementary Figure 2 [file 41419_2019_2137_MOESM2_ESM.png]

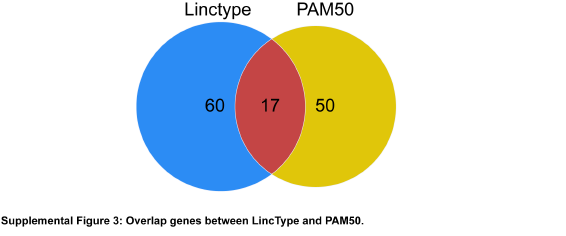

Supplement: Supplementary file 3 — Supplementary Figure 3 [file 41419_2019_2137_MOESM3_ESM.png]

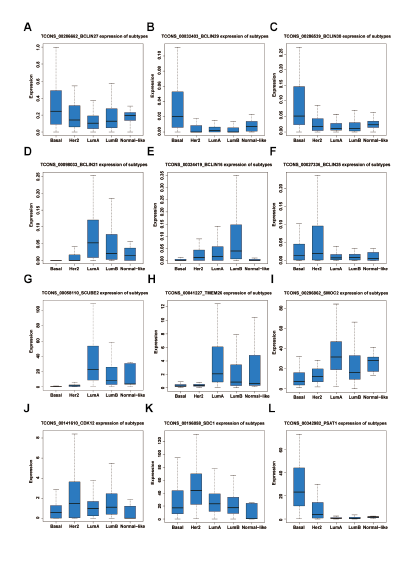

Supplement: Supplementary file 4 — Supplementary Figure 4 [file 41419_2019_2137_MOESM4_ESM.png]

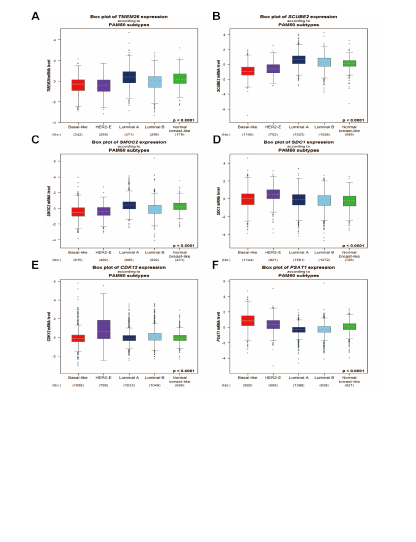

Supplement: Supplementary file 5 — Supplementary Figure 5 [file 41419_2019_2137_MOESM5_ESM.png]

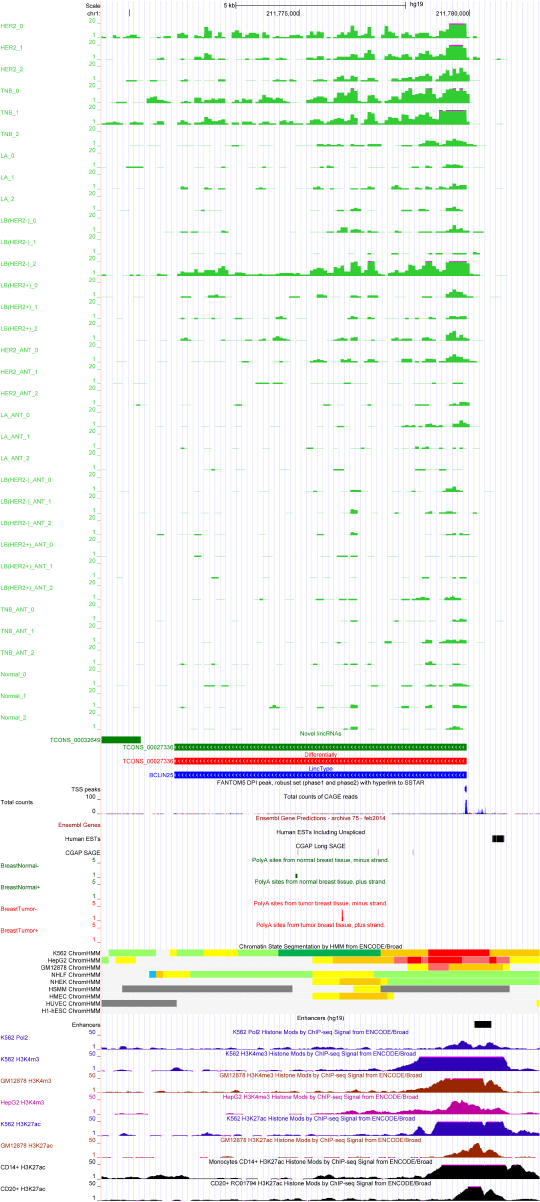

Supplement: Supplementary file 6 — Supplementary Figure 6 [file 41419_2019_2137_MOESM6_ESM.png]
