# Supplementary material for: The MS-lincRNA landscape reveals a novel lincRNA BCLIN25 that contributes to tumorigenesis by upregulating ERBB2 expression via epigenetic modification and RNA–RNA interactions in breast cancer
Source: Cell Death Dis. 2019 Dec 4;10(12):920. doi: 10.1038/s41419-019-2137-5 (PMC6892920; doi:10.1038/s41419-019-2137-5)
Supplement: Supplementary file 8 — Supplementary file legends [file 41419_2019_2137_MOESM8_ESM.doc]

**Additional file legends:**

**Additional file 1: Supplementary Tables**

Additional file 1: Table S1: PCR Primer and siRNA sequences.

Additional file 1: Table S2: Clinical information of the samples used in RNA-Seq.

Additional file 1: Table S3: Alignment result of RNA-Seq data by TopHat software.

Additional file 1: Table S4: Known genes and novel lincRNAs regulated by DNA methylation.

Additional file 1: Table S5: Functions of known genes of different modules in Figure 3B.

Additional file 1: Table S6: Details of the Linctype genes given in Figure 4A.

Additional file 1: Table S7: Potential microRNAs that binding the 3’-UTR of ERBB2 predicted in Targetscan, TarBase and RNA22 databases.

**Additional file 2: Supplementary Figures**

Additional file 2: Figure S1: Genes related to differentially expressed novel lincRNAs. (a) The number of associated genes per of 715 differentially expressed novel lincRNAs. (b) Distance of differentially expressed novel lincRNAs to transcriptional start site of related genes. (c) The number of differentially expressed novel lincRNAs related to previously characterized breast cancer genes.

Additional file 2: Figure S2: Workflow for Determination of Linctype genes.

Additional file 2: Figure S3: Overlap genes between LincType and PAM50.

Additional file 2: Figure S4: Validation of MSS-LincRNA in TCGA cohort. (a-c) MSS-LincRNA BCLIN27, BCLIN29, BCLIN30 were highly expressed in triple negative breast cancer. (d) MSS-LincRNA BCLIN21 was highly expressed in Luminal A subtype breast cancer. (e-f) MSS-LincRNA BCLIN16 was highly expressed in Luminal B subtype breast cancer and BCLIN25 in HER-2 subtype breast cancer. (g-i) SCUBE2, TMEM26, SMOC2 were highly expressed in Luminal A subtype breast cancer. (j-k) CDK12 and SDC1 were highly expressed in HER-2 subtype breast cancer. (i) PSAT1 was highly expressed in triple negative breast cancer.

Additional file 2: Figure S5: Linctype gene expression in GEO.

Additional file 2: Figure S6: Genome context of BCLIN25.

Additional file 2: Figure S7: (a) miR-133 expression with or without BCLIN25 knockdown in UACC-B12 cells. (b, c) Assignment of miR-125b mimic decreases the expression of ERBB2 mRNA (b) and protein level (c).
