# Supplementary material for: The MS-lincRNA landscape reveals a novel lincRNA BCLIN25 that contributes to tumorigenesis by upregulating ERBB2 expression via epigenetic modification and RNA–RNA interactions in breast cancer
Source: Cell Death Dis. 2019 Dec 4;10(12):920. doi: 10.1038/s41419-019-2137-5 (PMC6892920; doi:10.1038/s41419-019-2137-5)
Supplement: Supplementary file 16 — LANGUAGE EDITING CERTIFICATE [file 41419_2019_2137_MOESM16_ESM.pdf]

WILEY Wiley Editing Services

LANGUAGE EDITING CERTIFICATE

This document certifies that the manuscript listed below was edited for proper English language, grammar, punctuation, spelling, and overall style by one or more of the highly qualified native English speaking editors at Wiley Editing Services.

Manuscript title:

Landscape of MS-lincRNA identifies a novel lincRNA BCLIN25 contributing tumorigenesis via upregulation of ERBB2 expression by epigenetic modification and RNA-RNA interactions in breast cancer

Authors:

Shouping Xu1*, Hongbo Liu2*, Lin Wan1*, Weijia Zhang3, Qin Wang1, Shumei Zhang2, Shipeng Shang 2, Yan Zhang4, # & Da Pang1, 5, #

Date Issued:

February 22, 2019

Certificate Verification Key:

82F8-C07D-7418-B4F0-718P

This certificate may be verified at https://secure.wileyeditingservices.com/certificate. This document certifies that the manuscript listed above was edited for proper English language, grammar, punctuation, spelling, and overall style. Neither the research content nor the authors' intentions were altered in any way during the editing process. Documents receiving this certification should be English-ready for publication; however, the author has the ability to accept or reject our suggestions and changes. If you have any questions or concerns about this document or certification, please contact help@wileyeditingservices.com.

CERTIFIED Wiley Editing Services

Wiley Publishing Services is a service of Wiley Publishing. Wiley's Scientific, Technical, Medical, and Scholarly (STMS) business serves the world's research and scholarly communities, and is the largest publisher for professional and scholarly societies. Wiley is committed to providing high quality services for researchers. To find out more about Wiley Editing Services, visit wileyeditingservices.com. To learn more about our other author services provided by Wiley Publishing, visit authorservices.wiley.com.
