# Supplementary material for: The MS-lincRNA landscape reveals a novel lincRNA BCLIN25 that contributes to tumorigenesis by upregulating ERBB2 expression via epigenetic modification and RNA–RNA interactions in breast cancer
Source: Cell Death Dis. 2019 Dec 4;10(12):920. doi: 10.1038/s41419-019-2137-5 (PMC6892920; doi:10.1038/s41419-019-2137-5)
Supplement: Supplementary file 18 — DECLARATION OF CONTRIBUTIONS TO ARTICLE [file 41419_2019_2137_MOESM18_ESM.pdf]

DECLARATION OF CONTRIBUTIONS TO ARTICLE

ADMC

Manuscript Number: CDDIS-19-2694R

Journal Name: *Cell Death & Disease* (the 'Journal')

Proposed Title of the Contribution:

The MS-lincRNA landscape reveals a novel lincRNA BCLIN25 that contributes to tumorigenesis by upregulating ERBB2 expression via epigenetic modification and RNA-RNA interactions in breast cancer (the 'Contribution')

Author(s):

Shouping Xu, Hongbo Liu, Lin Wan, Weijia Zhang, Qin Wang, Shumei Zhang, Shipeng Shang , Yan Zhang, Da Pang (the 'Authors')

For all *CDDis* articles, each person named as an author in the published version must be able to show he or she has contributed substantially to the article.

Authorship credit should be based on 1) substantial contributions to conception and design, acquisition of data, or analysis and interpretation of data; 2) drafting the article or revising it critically for important intellectual content; and 3) final approval of the version to be published. Authors should meet conditions 1, 2 and 3.

Any person who cannot be shown to have made a substantial contribution to the article cannot be listed as an author in the final version. The name of any person who is deemed to have made a minor contribution can, however, appear in the Acknowledgments section of the article.

Please complete the table below to indicate the contributions of all named authors to the manuscript.

Author Full Name:	Specification of Contribution to the Manuscript:
Shouping Xu	substantial contributions to conception and design, drafting the article or revising it critically for important intellectual content
Hongbo Liu	acquisition of data, or analysis and interpretation of data
Lin Wan	acquisition of data, or analysis and interpretation of data
Weijia Zhang	acquisition of data, or analysis and interpretation of data
Qin Wang	acquisition of data, or analysis and interpretation of data
Shumei Zhang	acquisition of data, or analysis and interpretation of data
Shipeng Shang	acquisition of data, or analysis and interpretation of data
Yan Zhang	drafting the article or revising it critically for important intellectual content
Da Pang	substantial contributions to conception and design, final approval of the version to be published

Please complete the table below to indicate the contributions of all named authors to the figures.

Figure 1:

Shouping Xu,Hongbo Liu ,Yan Zhang and Da Pang

Figure 2:

Shouping Xu， Weijia Zhang ,Shipeng Shang and Da Pang

Figure 3:

Shouping Xu， Shumei Zhang ,Yan Zhang and Da Pang

Figure 4:

Shouping Xu， Shipeng Shang ,Yan Zhang and Da Pang

Figure 5:

Shouping Xu， Lin Wan,Qin Wang and Da Pang

Figure 6:

Shouping Xu， Lin Wan， Qin Wang and Da Pang

Signed for and on behalf of the Author(s):	Print Name:	Date:
Shouping Xu, Hongbo Liu	Shouping Xu, Hongbo Liu, Lin Wan, Weijia Zhang, Qin Wang, Shumei Zhang, Shipeng Shang , Ya	October 18, 2019

Lin Wan, Weijia Zhang Shipeng shang

Qin Wang Shumei Zhang Da Pang Yan Zhang
